# Surfactant‐Free Synthesis of Crystalline Mesoporous Metal Oxides by a Seeds/ NaCl‐Mediated Growth Strategy

**DOI:** 10.1002/advs.202304533

**Published:** 2023-11-08

**Authors:** Yuan Shu, Qian Liu, Meiyu Shi, Zequn Zhang, Chengmin Xie, Shuxian Bi, Pengfei Zhang

**Affiliations:** ^1^ State Key Laboratory of High‐efficiency Utilization of Coal and Green Chemical Engineering College of Chemistry and Chemical Engineering Ningxia University Yinchuan 750021 China; ^2^ School of Chemistry and Chemical Engineering Shanghai Jiao Tong University Shanghai 200240 China

**Keywords:** mechanochemistry, rod‐like nanoparticles, salt template, seeds‐mediated growth method, transitional metal oxides

## Abstract

Transitional metal oxides (TMOs) with ultra‐high specific surface areas (SSAs), large pore volume, and tailored exposed facets appeal to significant interests in heterogeneous catalysis. Nevertheless, synthesizing the metal oxides with all the above features is challenging. Herein, the so‐called seeds/NaCl‐mediated growth method is successfully developed based on a bottom‐up route. First, the (Brunauer‐Emmett‐Teller) BET SSAs of TMOs prepared with this method are significantly higher, where the BET SSAs of CeO_2_, SnO_2_, Nb_2_O_5_, Fe_3_O_4_, Mn_3_O_4_, Mg(OH)_2_, and ZrO_2_ reached 187, 275, 518, 212, 147, 186, and 332 m^2^ g^−1^, respectively. Second, these TMOs exhibit unique mesoporous structures, generated mainly by the aggregation of rod‐like or other aspherical primary nanoparticles. More importantly, no environmental‐unfriendly organic surfactants or expensive metal alkoxides are involved in this method. Therefore, the entire synthesis protocol fully fitted the “green synthesis” definition, and the corresponding TMOs prepares displayed excellent catalytic performance.

## Graphical abstract

Transitional metal oxides (TMOs) with ultra‐high specific surface areas (SSAs), large pore volume, and tailored exposed facets appeal to significant interests in heterogeneous catalysis. In this study, a green seeds/NaCl‐mediated growth method is successfully developed, where no surfactants or metal‐organic precursors are involved. Importantly, the prepared TMOs exhibit significantly improves SSAs and optimized mesoporous structure.

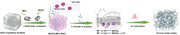

## 1 Introduction

Pore engineering of transitional metal oxides (TMOs) with the tailored pore structure appealed to significant attention in heterogeneous catalysis.^[^ ^1^, ^2^, ^3^ ^]^ In the state‐of‐art synthesis of porous TMOs (e.g., the sol‐gel methods,^[^ ^4^, ^5^ ^]^ the combustion methods,^[^ ^6^, ^7^ ^]^ surfactant‐assisted precipitation,^[^ ^8^ ^]^ and the template‐free self‐assembly method^[^ ^9^, ^10^, ^11^ ^]^), the pores inside TMOs mainly resulted from the stacking of primary metal oxide nanoparticles. Among them, due to an uneven particle size distribution of primary nanoparticles, the pore structures formed by primary particle accumulation are always disordered. Moreover, these primary nanoparticles often have a spherical morphology, which is of less interest in catalysis. Because the exposed {110} or {100} planes in nanorods were sometimes more catalytic active than {111} planes of nanoparticles. Typically, hard‐templating or hydrothermal methods were used to construct mesoporous TMOs with faceted primary nanoparticles.^[^ ^12^, ^13^ ^]^ However, the BET‐specific surface areas (SSAs) of TMOs prepared by these methods were often limited (e.g., Co_3_O_4_: 62 m^2^ g^−1^,^[^ ^14^ ^]^ CeO_2_: 121 m^2^ g^−1^,^[^ ^15^ ^]^ and NiO: 94 m^2^ g^−1^.^[^ ^16^ ^]^) In addition, these protocols often required time‐consuming steps (e.g., several days).^[^ ^17^, ^18^, ^19^ ^]^ To date, a synthetic principle for porous TMOs with non‐spherical primary nanoparticles should conform to low‐cost standards, environmental protection, simple process, and easy amplification.

As to the synthesis of metal oxide catalysts, the precipitation process was one of the most efficient unit operations. In view that the K_sp_ of transitional metal cations were normally <10^−14^ (e.g., K_sp_‐α‐Co(OH)_2_, Cu(OH)_2_, and Al(OH)_3_ = 1.09 × 10^−15^, 2.2 × 10^−20^, and 1.3 × 10^−33^ respectively),^[^ ^20^ ^]^ a high supersaturation of metal hydroxide in the solution was usually achieved. Besides, the distribution of the supersaturation ratio in the vessel during precipitation was often uneven with the location and time.^[^ ^21^ ^]^ These factors led to a quick and variable nucleation rate. Therefore, the TMO products by the precipitation were always poorly controlled. In contrast to the uncontrollable nucleation under the wet‐chemistry precipitation process, the solid‐state nucleation induced by mechanochemical activation could be carefully tuned by adjusting the grinding parameters: ball‐filling ratio, ball‐to‐powder mass ratio, and grinding time.^[^ ^22^, ^23^ ^]^ In addition, in the synthesis of colloidal nanoparticles, the secondary growth of initial seeds provided a great chance to produce a finer structure with a more uniform size and regular morphology.^[^ ^24^, ^25^, ^26^ ^]^ Inspired by the seed‐mediated method, the effective separation of nucleation and growth might be created by the combination of solvent chemistry and mechanochemistry.^[^ ^27^ ^]^ The whole synthetic process was designed in two steps: TMO seed synthesis by the solid‐state ball milling process and subsequent seed growth under a solution environment. Therefore, it would be interesting to figure out whether combining solid‐state mechanochemistry and wet chemistry could control the structure of TMOs.

Different from the previous literature that TMOs were only produced by solid‐state precipitation,^[^ ^22^, ^28^ ^]^ in this paper we established a so‐called seed/NaCl‐mediated growth method consisting of two steps: the first step of solid‐state precipitation and the second step of hydrolysis or precipitation in solution. Specifically, the nanoparticles supported on NaCl after the solid‐state precipitation served as seeds. Moreover, the disappearance of the TMO‐NaCl interface during solution growth exposed an unsaturated crystal surface that was originally stabilized by NaCl. These highly reactive crystal surfaces with unsaturated coordination provided possible reaction sites for subsequent seed growth. Interestingly, after the secondary growth, a series of mesoporous metal oxides with ultra‐high SSAs were successfully prepared (e.g., SnO_2_: 275, Nb_2_O_5_: 495, ZrO_2_: 332, Fe_3_O_4_: 212, Mn_3_O_4_: 147, CeO_2_: 187, and Mg(OH)_2_: 186 m^2^ g^−1^). To our best knowledge, the BET SSAs of these metal oxides kept the records in the absence of using surfactants or organic metal salts. More interestingly, primary nanoparticles inside the mesoporous TMOs were partially exposed to {100} or {110} planes rather than traditional {111} facets. Due to the high BET SSA, large pore volume, and unsaturated lattice planes, the corresponding TMO, e.g., NiO‐Co_3_O_4_, exhibited an excellent methane redox performance.

## 2 Results and Discussions

**Figure 1:**
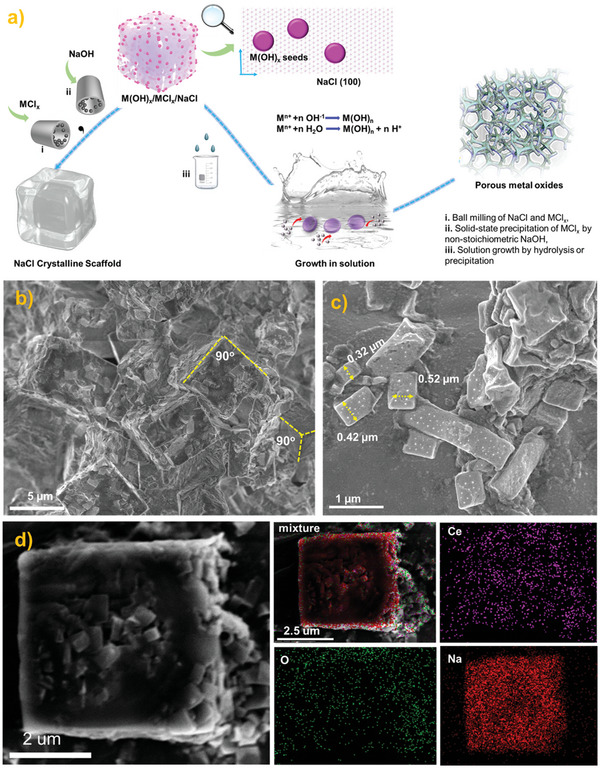
The process for building the structure of Ce(OH)_3_ seeds on NaCl. a) Scheme of Seeds/NaCl‐mediated growth method for porous TMOs synthesis. b) SEM image of Ce(OH)_3_‐CeCl_3_‐NaCl after the second ball‐milling. c) SEM image of Ce(OH)_3_‐CeCl_3_‐NaCl under a higher magnification. d) The EDS mappings of a selected cubic NaCl nanoparticle.

Taking advantage of the cubic morphology of micron‐size NaCl crystals, previous studies showed that NaCl could effectively serve as a template to tailor product morphology.^[^ ^29^, ^30^ ^]^ Herein, the role of NaCl crystals as a solvent, support, and “structure directing agent” was discovered, and a so‐called seeds/NaCl‐mediated growth method was developed based on this. To elucidate the seeds/NaCl‐mediated growth method, the synthesis of porous CeO_2_ was selected as the first example. As displayed in **Figure** **1a**, before the solid‐state nucleation by precipitation reaction, metal chloride salts, e.g., CeCl_3_ should be ball‐milled with NaCl crystals to achieve a high dispersion, which was a prerequisite to produce small‐size seeds according to the previously reported literature. As displayed in the XRD diffraction pattern (Figure S1a, Supporting Information), a high dispersion of CeCl_3_ (2 mmol) on the NaCl crystalline scaffold was well obtained after the first ball milling, where only NaCl characteristic peaks could be seen. In addition, part of CeCl_3_ would enter into the NaCl lattice because a significant shift of NaCl diffraction peaks toward a higher angle was observed (Figure S1b, Supporting Information). However, a mixture phase of CeCl_3_ and KCl was presented when using KCl as a salt template (Figure S1c, Supporting Information), further indicating NaCl was the suitable scaffold to disperse transitional metal chloride salt. The high dispersion of CeCl_3_ over NaCl nanoparticles was also demonstrated by SEM images shown in Figure S2 (Supporting Information), where NaCl nanoparticles with a regular cubic morphology were uniformly coated by a layer of amorphous CeCl_3_. Then, after adding 2 mmol NaOH with a non‐stoichiometric ratio, one‐third of the CeCl_3_ salt would react with NaOH to produce Ce(OH)_3_ seeds by the solid‐state precipitation reaction. Interestingly, no characteristic peaks attributed to Ce(OH)_3_ or CeO_2_ could be found after the solid precipitation (Figure S1a, Supporting Information), showing that the formed Ce(OH)_3_ or CeO_2_ seeds were small in size and evenly dispersed on the surface of NaCl. Therefore, a mixture of Ce(OH)_3_ seeds and CeCl_3_ would co‐exist on the NaCl crystalline scaffold as Ce(OH)_3_‐CeCl_3_‐NaCl after the grinding.

SEM equipped with an EDS detector was then utilized to carefully analyze the final morphology of NaCl, and the dispersion of Ce(OH)_3_ seeds after the second grinding. As seen in Figure 1b, due to a low Moh's hardness of NaCl, the NaCl nanoparticles that had experienced the first ball grinding could still be further broken down. The original cubic NaCl nanoparticles in Figure S2 (Supporting Information) with ≈10 µm were now reduced to 5 µm. In addition, some small‐size NaCl fragments from 0.3–0.5 µm were also found in the fracture surface of the large NaCl nanoparticle (Figure 1c). Interestingly, these NaCl nanoparticles still exhibited dominated {100} planes, indicating NaCl could serve as a stable cubic mound to optimize product morphology. EDS mapping was then analyzed to disclose the dispersion of Ce(OH)_3_ seeds on NaCl. As displayed in Figure 1d, signals of Na, Ce, and O were full coverage of the selected cubic nanoparticle, demonstrating the high dispersion of Ce(OH)_3_ seeds was achieved over the {100} facets of NaCl after the second ball milling. The existence of NaCl also stabilized the Ce(OH)_3_‐NaCl interface and avoided the aggregation of Ce(OH)_3_ seeds. In addition, once the removal of NaCl, the NaCl‐Ce(OH)_3_ interface disappeared, thus exposing the originally closed Ce(OH)_3_ facet for subsequent growth in solution. Generally, NaCl crystals actually worked well as solvent, seeds carrier, and capping agent.

**Figure 2:**
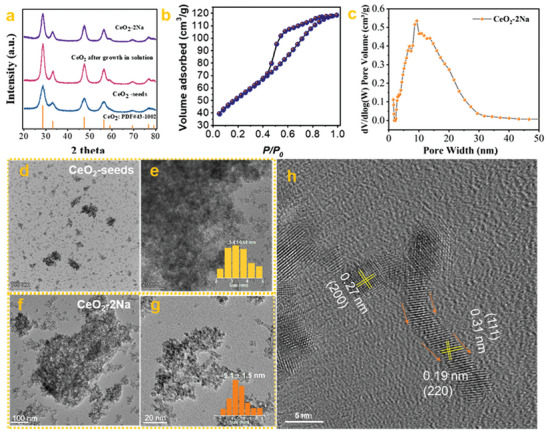
The evidence for the seeds/NaCl‐mediated growth method. a) the XRD patterns of CeO_2_ seeds before and after growth. b) N_2_ adsorption curve of CeO_2_‐2Na. c) the pore size distribution of CeO_2_‐2Na. d,e) TEM images of CeO_2_‐seeds with different magnifications. f,g) TEM images of CeO_2_‐2Na with different magnifications. h) HRTEM image of CeO_2_‐2Na.

Subsequently, a wet chemistry hydrolysis process was induced to provide a favorable seed‐mediated growth environment. Theoretically, the supported Ce(OH)_3_ seeds and the unreacted metal chlorine salts (CeCl_3_) were immediately released into an aqueous solution after dissolving the NaCl template. Meanwhile, the precipitation of unreacted CeCl_3_ would happen over the neighborhood Ce(OH)_3_ seeds by dropwise addition of NaOH solution. Therefore, the unreacted CeCl_3_ on NaCl crystalline was expected to play a stock‐solution‐like role, which further provided the monomers for subsequent seed growth in an aqueous solution. In addition, a large number of unsaturated crystalline facets of Ce(OH)_3_ seeds after removing the TMO‐NaCl interface would be exposed. Therefore, an orientation growth of CeCl_3_ over Ce(OH)_3_ seeds might occur. The out situ XRD was carefully used to monitor the growth of Ce(OH)_3_ seeds in the solution. As seen in **Figure** **2**a, the Ce(OH)_3_ seeds exhibited a typical CeO_2_ crystalline, indicating rapid oxidation of Ce(OH)_3_ to CeO_2_ during ball milling. Therefore, the preclaimed Ce(OH)_3_ seeds were actually CeO_2_ seeds.

Compared with the XRD pattern of CeO_2_ seeds, the full widths at half minimum (FHWM) of CeO_2_ after growth and calcination increased enormously, indicating a clear growth of CeO_2_ nanoparticles in size. Specifically, according to the Sheller calculation, the calculated average crystalline size of CeO_2_‐growth and CeO_2_ seeds was 5.8 and 3.7 nm, respectively. In addition, the peak intensity ratios of the (111) to (200) peaks [I(200)/I(111)] decreased from 4.24 in the CeO_2_ seeds to 3.33 in the CeO_2_‐2Na, suggesting the growth of (200) orientation were promoted by the growth in solution. More importantly, CeO_2_ prepared by this seeds/NaCl‐mediated growth method exhibited an ultra‐high BET SSA. Notably, the BET SSA of the optimized CeO_2_‐2Na sample reached 189 m^2^ g^−1^, which was nearly two folds of the CeO_2_ prepared by the previous solid‐state NaCl method (Figure 2b and **Table** **1**).^[^ ^22^ ^]^ Additionally, the pore size distribution of CeO_2_‐2Na calculated by the nonlocal density functional theory model (NLDFT) was mainly located in the range of ≈0–30 nm, indicating the formation of a mesoporous structure within CeO_2_‐2Na (Figure 2c).

**Table 1:** The texture of porous metal oxides prepared by these seeds/NaCl‐mediated methods.

Sample	_mmol (MClx)	NaOH amount	SSA[m^2^ g^−1^]	d [nm]^a)^
CeO_2_‐2Na	2 mmol(CeCl_3_)	2 mmol	189	7.2
CeO_2_‐NaCl^b)^	2 mmol(CeCl_3_)	6 mmol	84	5.56
Mn_3_O_4_‐1Na	2 mmol(MnCl_2_)	1 mmol	147	13.6
Mn_3_O_4_‐NaCl^b)^	2 mmol(MnCl_2_)	4 mmol	79	17.3
Nb_2_O_5_‐1Na	1 mmol(NbCl_5_)	1 mmol	495	2.8
Nb_2_O_5_‐NaCl^b)^	1 mmol(NbCl_5_)	5 mmol	62	16.3
Fe_3_O_4_‐2Na	2 mmol(FeCl_3_)	2 mmol	212	3.6
Fe_3_O_4_‐NaCl^b)^	2 mmol(FeCl_3_)	6 mmol	141	12.3
Mg(OH)_2_‐1Na	2 mmol(MgCl_2_)	1 mmol	186	11.3
Mg(OH)_2_‐NaCl^b)^	2 mmol(MgCl_2_)	4 mmol	76	15.1
SnO_2_‐1Na	2 mmol(SnCl_2_)	1 mmol	276	3.9
SnO_2_‐NaCl^b)^	2 mmol(SnCl_2_)	4 mmol	83	6.7
ZrO_2_‐2Na	2 mmol(ZrCl_4_)	2 mmol	332	3.5
ZrO_2_‐2Na^b)^	2 mmol(ZrCl_4_)	8 mmol	152	6.5

^a)^ The average pore diameter calculated by the BJH model;

^b)^ The materials synthesized by the previously reported NaCl method.

The evolution of CeO_2_ seeds morphology and size was then carefully investigated by TEM and HRTEM. As shown in Figure 2d,e, many small and dissociative CeO_2_ nano aggregates after the dissolution of the NaCl crystalline scaffold were observed. Based on the above 50 random CeO_2_ nanoparticles in Figure 2d, the average size of CeO_2_ seeds was calculated as 3.1 nm, which was consistent with the calculation from the XRD (Figure 2a). The dominant facet of CeO_2_ seeds was {111} facets according to Figure S3 (Supporting Information), where the interplanar spacing was almost 0.31 nm. Besides the {111} facets, the {200} facets with interplanar spacing of 0.27 nm were also presented, which might be the interface of CeO_2_‐cubic NaCl before water washing. Interestingly, the morphology of CeO_2_ changed enormously after the growth in solution, original well‐dispersed CeO_2_ nanoparticles disappeared, and giant CeO_2_ nano‐aggregates occurred as the dominant morphology. Such a nanostructure was just like an interesting “Lego” building block, where the stack of CeO_2_ nanoaggregate made the final CeO_2_ rich with internal pores. With further observation in Figures 2f,g, the morphology and size of CeO_2_ nanoparticles after growth changed greatly. First, the average size of CeO_2_ after growth rapidly increased to 5.6 nm. Second, some CeO_2_ nanoparticles with worm‐like shape were observed. Specifically, clear worm‐like CeO_2_ nanoparticles were presented in Figure 2h, indicating a possible oritention growth of CeO_2_ seeds. In general, porous CeO_2_ with advantages in SSA and morphology are simultaneously synthesized by this seeds/NaCl‐mediated growth method.

**Figure 3:**
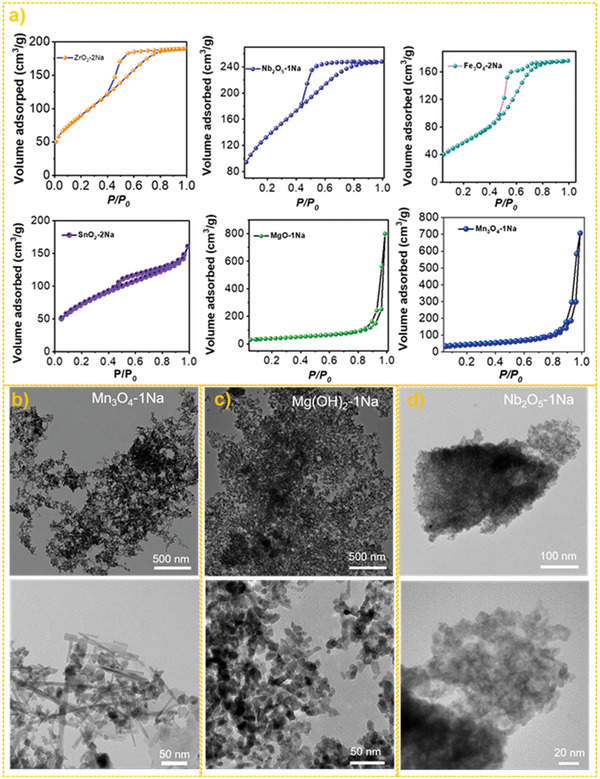
The texture of porous metal oxides by this seeds/NaCl‐mediated method. a) N_2_ adsorption curves of prepared porous TMOs, b) TEM images of Mn_3_O_4_‐2Na, c) TEM images of Mg(OH)_2_‐1Na, d) TEM images of Nb_2_O_5_‐1Na.

Notably, this seed/NaCl‐mediated growth method for producing mesoporous or porous TMOs was a general principle that either hydrolyzed, weakly hydrolyzed, or nonhydrolyzed metal precursor salts were readily synthesized to the corresponding oxides, such as SnO_2_, Fe_3_O_4_, Nb_2_O_5_, ZrO_2_, Mn_3_O_4,_ and Mg(OH)_2_. Specifically, the BET SSAs of SnO_2_, Fe_3_O_4_, Nb_2_O_5_, ZrO_2_, Mn_3_O_4_ and Mg(OH)_2_ could reach 276, 212, 495, 332, 147, and 186 m^2^ g^−1^ (Table 1 and **Figure** **3**a). A comprehensive comparison of these BET SSAs with literature was listed in Table S1 (Supporting Information), according to the results, the BET SSAs of porous metal oxides prepared by this method reached record values in the absence of using surfactants or organic metal salts.^[^ ^31^, ^32^, ^33^, ^34^, ^35^ ^]^ Although all the samples exhibited an evident XRD peak‐broadening phenomenon due to the small grain sizes, these metal oxide products still exhibited clear XRD diffiraction peaks (Figure S4, Supporting Information). Besides the ultra‐high BET surface areas, the synthesized oxides, e.g., Mn_3_O_4_, Mg(OH)_2_, and Nb_2_O_5_ also exhibited an advantage in morphology. According to Figure 3b, the Mn_3_O_4_‐1Na exhibited a particularly dendritic spine morphology. With further observation, many rod‐like Mn_3_O_4_ nanoparticles were found in the Mn_3_O_4_‐1Na, which might act as the “dendritic branches” to block the proximity of neighboring nanoparticles. In addition, the occurrence of Mn_3_O_4_ nanorods was also the trace for seed growth, because the morphology of Mn_3_O_4_ seeds was spherical (Figure S5, Supporting Information). Similarly, in the Mg(OH)_2_ sample, many Mg(OH)_2_ nanorods with a small aspect ratio were also clearly found in the TEM, which then assembled into a reef‐like morphology. TEM images of Nb_2_O_5_ also exhibited a spongy‐like microstructure with the interior filled with voids. The above facts strongly supported the effectiveness and universality of this seeds/NaCl‐mediated method.

**Figure 4:**
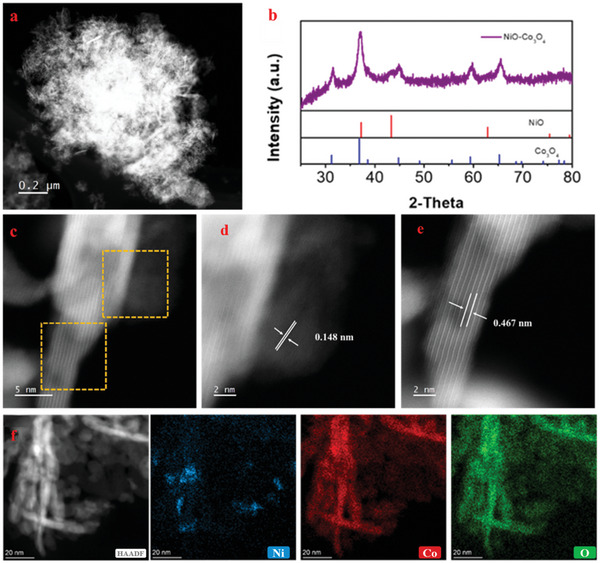
The characterization of hybrid NiO‐Co_3_O_4_‐seed. a) TEM image of the NiO‐Co_3_O_4_‐seed. b) the XRD diffraction of NiO‐Co_3_O_4_‐seed. c–e) Cs‐corrected HRTEM images of NiO‐Co_3_O_4_‐seed. f) HAADF image and EDS elemental mappings of NiO‐Co_3_O_4_‐seed.

Typically, due to the significant differences in energy dispersive spectroscopy, the case of CoCl_2_ grown on hetero‐NiO seeds was then investigated, and the corresponding sample was named NiO‐Co_3_O_4_‐seed. As displayed in **Figure** **4**a, many needle‐like nanoparticles rather than spherical ones appeared in the NiO‐Co_3_O_4_‐seed sample, which was quite different from the Co_3_O_4_ or NiO nanoparticles prepared by the NaCl‐template method.^[^ ^22^, ^36^ ^]^ The XRD diffraction of the product displayed both the Co_3_O_4_ and NiO characteristic peaks, indicating a co‐existence of NiO and Co_3_O_4_ species (Figure 4b). To further demonstrate whether Co_3_O_4_ grew on heterogeneous NiO seeds, Cs‐corrected HRTEM images and EDS elemental mappings of NiO‐Co_3_O_4_‐seed were carefully collected. As shown in Figure 4c–e, a thin nanorod and a nanoparticle attached to the side of the nanorod were exhibited. Empirically, the smaller nanoparticle with hemispherical morphology was possible as NiO seeds because the particles produced by the ball milling process were often spherical,^[^ ^22^ ^]^ while the nanorod with well‐controlled morphology was suggested as Co_3_O_4_. Besides, the lattice spacing of 0.15 nm measured in the hemispherical nanoparticle was attributed to the NiO (220) plane,^[^ ^37^ ^]^ while the lattice spacing of 0.47 nm in the nanorod belonged to Co_3_O_4_ (111) planes (Figure 4c).^[^ ^38^ ^]^ Therefore, the nanoparticle and nanorod presented in Figure 4c were NiO and Co_3_O_4_, respectively. The EDS elemental analysis of NiO‐Co_3_O_4_‐seed was further adopted to demonstrate this hypothesis. According to Figure 4d, the EDS signal of Ni was only concentrated on nanoparticles with small sizes, while the Co signal was full coverage of the selected region. Accordingly, combined with the Cs‐HRTEM images and EDS elemental mappings, a possible surface deposition of Co_3_O_4_ over Ni(OH)_2_ occurred, where pre‐existing Ni(OH)_2_ nanoparticles with strong basicity might promote the accumulation of dissociated Co^2+^ cations to the periphery of Ni(OH)_2_ particles, and the subsequent precipitation of Co^2+^ cation would occur across the high‐index surface of NiO, e.g., the (220) planes.

**Figure 5:**
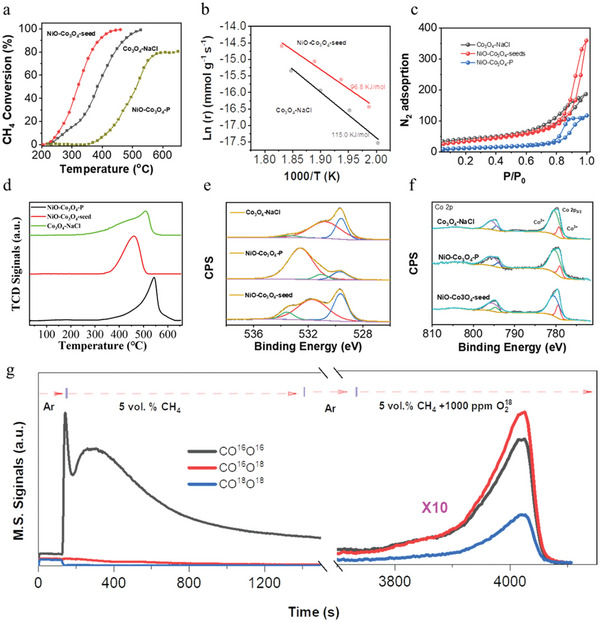
The catalytic performance of NiO‐Co_3_O_4_‐seed. a) Light‐off curves of CH_4_ combustion over NiO‐Co_3_O_4_‐seed, Co_3_O_4_‐NaCl, and NiO‐Co_3_O_4_‐P under the WHSV of 40,000 ml g^−1^ s^−1^. b) the CH_4_ catalytic combustion activation energy of NiO‐Co_3_O_4_‐seed, and Co_3_O_4_‐NaCl. c) N_2_ adsorption curves of NiO‐Co_3_O_4_‐seed, NiO‐Co_3_O_4_‐P, and Co_3_O_4_‐NaCl. d) the CH_4_‐TPR curves of NiO‐Co_3_O_4_‐seed, NiO‐Co_3_O_4_‐P, and Co_3_O_4_‐NaCl. e) O 1s XPS spectra of NiO‐Co_3_O_4_‐seed, NiO‐Co_3_O_4_‐P, and Co_3_O_4_‐NaCl. f) Co 2p XPS spectra of NiO‐Co_3_O_4_‐seed, NiO‐Co_3_O_4_‐P, and Co_3_O_4_‐NaCl. g) Time‐dependent mass spectra of C^16^O^16^O, C^16^O^18^O, and C^18^O^18^O species during the ^18^O_2_ isotope labeling experiment over NiO‐Co_3_O_4_‐seed at 280 °C.

Compared with the bulk metal oxides without pores, TMOs nanoparticles with rich porosity often exhibited a much‐improved redox performance. Here, having the features of ultra‐high exposed surface areas and particular rod‐like morphologies, TMOs synthesized by this method (e.g., NiO‐Co_3_O_4_‐seed) would serve well as an effective catalyst to ignite the corresponding chemical reactions. Next, the CH_4_ catalytic combustion reaction was used to study the redox ability of NiO‐Co_3_O_4_‐seed. In addition, the samples of NiO‐Co_3_O_4_‐P, Co_3_O_4_‐NaCl, Co_3_O_4_‐seed and Co_3_O_4_‐NiO‐seed were selected as the contrast samples (details seen in the section of Methods). Among the five catalysts, NiO‐Co_3_O_4_‐seed exhibited an excellent CH_4_ combustion performance. 50% CH_4_ completely burned into CO_2_ over NiO‐Co_3_O_4_‐seed occurred just at 322 °C, while the T_50_ over Co_3_O_4_‐seed, Co_3_O_4_‐NiO‐seed, Co_3_O_4_‐NaCl, and NiO‐Co_3_O_4_‐P was 358, 374, 383, and 510 °C, respectively (**Figure** **5**a). Moreover, as shown in the Figure 5b, the calculated CH_4_ catalytic combustion activation energy (Ea) of NiO‐Co_3_O_4_‐seed was 96.8 KJ mol^−1^, much lower than that of Co_3_O_4_‐NaCl (115.0 KJ mol^−1^).

The morphology and structure of spent NiO‐Co_3_O_4_‐seed after the CH_4_ combustion test were also explored by TEM and XRD. According to Figure S6a (Supporting Information), the spent NiO‐Co_3_O_4_‐seed still exhibited a mixture of NiO and Co_3_O_4_ characteristic diffraction peaks, indicating no diffusion of Ni^2+^ into the Co_3_O_4_ lattice after the high‐temperature reaction. Besides, as shown in Figure S6b–d (Supporting Information), a hybrid nanorod and nanoparticles morphology of spent NiO‐Co_3_O_4_‐seed was retained, indicating this particular hybrid nanorod and nanoparticles morphology of NiO‐Co_3_O_4_‐seed was also thermally stable. Finally, a long‐term thermal stability testing of NiO‐Co_3_O_4_‐seed was performed under 300 °C. As shown in Figure S7 (Supporting Information), no slight deactivation of NiO‐Co_3_O_4_‐seed was observed (the CH_4_ conversion at the 1^st^ h was 36.5% while the CH_4_ conversion at the 20^th^ h was 29.8%). In addition, as displayed in Figure S8 (Supporting Information), no evident sintering of NiO nanoparticles or Co_3_O_4_ nanorods was observed over NiO‐Co_3_O_4_‐seed after the 20 h stability test. Therefore, the synthesized NiO‐Co_3_O_4_‐seed could act well as a robust redox catalyst.

As shown in Figure 5b, N_2_ adsorption indicated a similar BET SSAs of NiO‐Co_3_O_4_‐seed (121 m^2^/g) and Co_3_O_4_‐NaCl (130.6 m^2^ g^−1^), but the BET SSA of NiO‐Co_3_O_4_‐P was quite small only 44.0 m^2^ g^−1^. In addition, according to XPS fitting results (Figure S9, Supporting Information), the Cl^−1^ residues on NiO‐Co_3_O_4_‐seed, Co_3_O_4_‐NaCl, and NiO‐Co_3_O_4_‐P were calculated as 0.55, 0.86, and 6.18 atom %, respectively. Therefore, the sample of Co_3_O_4_‐NaCl was a suitable contrast sample to understand the higher activity of NiO‐Co_3_O_4_‐seed. H_2_‐TPR, CH_4_‐TPR, and XPS were further used to characterize the differences among NiO‐Co_3_O_4_‐seed, Co_3_O_4_‐NaCl, and NiO‐Co_3_O_4_‐P. Higher oxygen vacancy concentration and lower oxygen vacancy formation energy were always observed in samples of Co_3_O_4_ nanocubes or nanorods.^[^ ^39^, ^40^ ^]^ According to the data of H_2_‐TPR (Figure S10, Supporting Information), CH_4_‐TPR (Figure 5d), O1s XPS (Figure 5e) and Co 2p XPS (Figure 5f), the NiO‐Co_3_O_4_‐seed indeed exhibited the lowest H_2_ and CH_4_ reduction temperature, the highest oxygen vacancies concentration (O_ads_/O_lat_ = 1.71), and the highest Co^3+^ concentration (Co^3+^/Co^2+^ = 0.25) among the three catalysts.

Finally, the CH_4_ catalytic mechanism over this NiO‐Co_3_O_4_‐seed was also glanced at by the O^18^ isotope labeling experiment. As shown in Figure 5g, after switching the Ar gas flow into 5 vol.% CH_4_, the signals of C^16^O^16^O immediately appeared and no C^16^O^18^O or C^18^O^18^O signals were observed. As the reaction time increased, the intensity of C^16^O^16^O tended to decrease due to continuous consumption of active lattice oxygen atoms or active adsorped oxygen species. To further explore which oxygen species participated in the combustion, a 5 vol.% CH_4_ mixture with a 1000 ppm ^18^O_2_ gas was then fed into the reaction. At the early stage (3700s–3900s), the main CO_2_ signals were composed of C^16^O^16^O and C^16^O^18^O, and no significant C^18^O^18^O M.S. signals were found. The presence of evident C^16^O^18^O signals indicated that the gaseous ^18^O_2_ proceeded the steps of adsorption (^18^O_2, ads_^δ−^), dissociation (^18^O_2_^2−^, ^18^O^2−^), and the reaction, rather than the pathway that CH_4_ directly reacted with the adsorbed oxygen. However, further increasing reaction time, the intensity of C^18^O^18^O would also increase due to the continuous depletion of ^16^O lattice oxygen atoms. The ^18^O_2_ isotope labeling experiments demonstrated the CH_4_ combustion over NiO‐Co_3_O_4_‐seed followed a Mars‐van Kre1velen mechanism, where the oxygen vacancies concentration played a vitally important role. Therefore, the primary Co_3_O_4_ nanorods in the NiO‐Co_3_O_4_‐seed with the higher oxygen vacancies concentration were responsible for the excellent performance of the NiO‐Co_3_O_4_‐seed.

## 3 Conclusion

In summary, through a combination of mechanochemistry and wet chemistry, we have successfully developed a seeds/NaCl‐mediated growth protocol to fabricate porous TMOs. Interestingly, it was found that both the advantages of mechanochemistry and wet chemistry were well utilized. The ball milling process was helpful to construct seeds with much smaller sizes, and the growth of the seeds in the solution provided good morphology control. In this regard, highly porous metal meanwhile with good crystalline with a high SSA (e.g, SnO_2_: 275, Nb_2_O_5_: 495, ZrO_2_: 332, Fe_3_O_4_: 212, Mn_3_O_4_: 147, CeO_2_: 187, and Mg(OH)_2_: 186 m^2^ g^−1^) could be easily prepared. More importantly, these porous TMOs synthesized were found to have rod‐like, worm‐like, or sheet‐like primary nanoparticles. These non‐spherical nanoparticles functioned well as seeds and were further assembled into a porous network. Additionally, the prepared NiO‐Co_3_O_4_‐seed exhibited a robust CH_4_ catalytic combustion performance comparable to NiO‐Co_3_O_4_‐NaCl and Co_3_O_4_‐NaCl. All in all, this surfactant‐free seeds/NaCl‐mediated growth strategy could offer a new opportunity for simply and low‐costly preparing porous TMOs in the future.

## Conflict of Interest

The authors declare no conflict of interest.

## Author Contributions

Y.S. and Q.L. contributed equally to this work. The manuscript was written through the contributions of all authors. All authors have approved the final version of the manuscript.

## Supporting information

Supporting Information

## Data Availability

The data that support the findings of this study are available from the corresponding author upon reasonable request.
