# Supplementary material for: Surfactant‐Free Synthesis of Crystalline Mesoporous Metal Oxides by a Seeds/ NaCl‐Mediated Growth Strategy
Source: Adv Sci (Weinh). 2023 Nov 8;11(1):2304533. doi: 10.1002/advs.202304533 (PMC10767421; doi:10.1002/advs.202304533)
Supplement: Supplementary file 1 — Supporting Information [file ADVS-11-2304533-s001.pdf]

ADVANCED SCIENCE
Open Access

Supporting Information

for *Adv. Sci.*, DOI 10.1002/advs.202304533

Surfactant-Free Synthesis of Crystalline Mesoporous Metal Oxides by a Seeds/NaCl-Mediated Growth Strategy

Yuan Shu, Qian Liu, Meiyu Shi, Zequn Zhang, Chengmin Xie, Shuxian Bi and Pengfei Zhang**

Supporting Information

Surfactant-free Synthesis of Crystalline Mesoporous Metal Oxides by A Seeds/ NaCl-mediated Growth Strategy

Yuan Shu,* Qian Liu, Meiyu Shi, Zequn Zhang, Chengmin Xie, Shuxian Bi, Pengfei Zhang*

Table of Contents

Figure S1. XRD patterns of NaCl and $CeCl_3$ during the solid-state precipitation....5
Figure S2. The SEM images of $CeCl_3$-NaCl after the first ball-milling....6
Figure S3. The HRTEM of CeO_2 seeds....6
Figure S4. XRD patterns of the prepared TMOs....7
Figure S5. TEM image of Mn_3O_4 seeds....7
Figure S6. XRD pattern and TEM of spent NiO-Co_3O_4-seeds....8
Figure S7 The stability testing of NiO-Co_3O_4-seed under 300℃....9
Figure S8. The TEM images of NiO-Co_3O_4-seed after 20 h stability testing....9
Figure S9. The XPS surveys of NiO-Co_3O_4-seed, NiO-Co_3O_4-P and Co_3O_4-NaCl....10
Figure S10. H2-TPR curves of NiO-Co_3O_4-seed, NiO-Co_3O_4-P and Co_3O_4-NaCl....10
Table S1. Comparison of the BET SSAs of TMOs with literature....11

Experimental Procedures

Chemical materials.

NaCl (Aladdin Reagent, ≥99.5%), anhydrous $SnCl_2$ (Energy Chemical Reagent, ≥98%), anhydrous $MnCl_2$ (Energy Chemical Reagent, ≥98%), anhydrous $FeCl_3$ (Aladdin Reagent, ≥99.5%), anhydrous $CeCl_3$ (Aladdin Reagent, ≥99.5%), anhydrous $ZrCl_4$ (Energy Chemical Reagent, ≥98%), anhydrous $MgCl_2$ (Aladdin Reagent, ≥99.5%), anhydrous $NbCl_5$ (Aladdin Reagent, ≥99%), and granular NaOH (Aladdin Reagent, ≥98%), were used without further purification.

Catalyst Preparation

Synthesis of mesoporous CeO_2-2Na

In the solid-state nucleation step (I), a mixture of 1 g NaCl and 2 mmol $CeCl_3$ was added to a 50 ml stainless steel ball mill jar, which contained four large stainless-steel balls (diameter: 1.2 cm) and twelve small stainless-steel balls (diameter: 0.4 cm). The ball-milling jar was then sealed, transferred to a three-dimensional high-speed vibrating ball mill machine (MSK-SFM-3), and ran for 0.5 h. Subsequently, 2 mmol of non-stoichiometric NaOH solid was added to the above mixture, and the grinding was continued

for another 0.5 h. In the solution-growth step, the product after the second ball milling was transferred to a round-bottomed flask and quickly dissolved in 50 ml of deionized water. After 5 min of sonication, 0.5 mol/L NaOH solution was slowly dropped into the solution under vigorous magnetic stirring until the pH became 9. The stirring of the solution was continued for 30 min. After the precipitate was washed three times by centrifugation, the sample was dried at 80℃ for 24 h. The final CeO_2-2Na was produced by calcined at 300℃ for 1 hour. The resulting CeO_2 sample based on the amount of NaOH was named as CeO_2-2Na, where the CeO_2-xNa indicated that x mmol NaOH was used in the ball-milling step. The synthesis steps of Mn_3O_4, Co_3O_4, $Mg(OH)_2$, and Fe_3O_4 were the same as those of Mn_3O_4.

Synthesis of mesoporous SnO_2

A mixture of 1 g NaCl and 2 mmol $SnCl_2$ was together added to a 50 ml stainless steel ball milling jar with the same milling parameters as above. The ball-milling jar was then sealed, transferred to the MSK-SFM-3 machine, and ball-milled for 0.5 h. After the first grinding, 1 mmol of non-stoichiometric NaOH solid was added to the above mixture, and then ball milling was continued for another 0.5 h. In the solution growth period, the above ball-milled mixture was quickly dispersed in 20 ml of ethanol. Then the 50 ml deionized H_2O was slowly added into the above solution to induce the hydrolysis of $SnCl_2$. To fully hydrolyze $SnCl_2$, the above solution was then stirred at room temperature for 1 h. After the precipitate was washed three times by centrifugation, the final SnO_2-1Na was obtained after drying and calcination at 300°C for 2 h. The synthetic steps of ZrO_2-2Na, Nb_2O_5-1Na were the same as SnO_2-1Na.

Synthesis of porous NiO-Co_3O_4-seed

Firstly, a mixture of 1 g NaCl and 1 mmol $NiCl_2$ was added to the ball milling reactor and the milling parameters were kept unchanged. Different from the above growth process, 2 mmol of stoichiometric NaOH was added to fully participate in $NiCl_2$. In the solution growth period, the above NiO-NaCl mixture was quickly dispersed into 100 ml $CoCl_2$ solution (0.03 mol/L) and sonicated for 5 min. Then, the 0.5 mol/L NaOH solution was dropped into the above solution under strong magnetic stirring until the PH of the solution was 9. The stirring of the solution was continued for 30 min. After the precipitate was washed

three times by centrifugation, the dried product was calcined at 300°C for 2 h to give the hybrid NiO-Co_3O_4 nanostructure.

Synthesis of porous Co_3O_4-NiO-seed

Firstly, a mixture of 1 g NaCl and 1 mmol $CoCl_2$ was added to the ball milling reactor and the milling parameters were kept unchanged. Different from the above growth process, 2 mmol of stoichiometric NaOH was added to fully participate in $CoCl_2$. In the solution growth period, the above $Co(OH)_2$-NaCl mixture was quickly dispersed into 100 ml $NiCl_2$ solution (0.03 mol/L) and sonicated for 5 min. Then, the 0.5 mol/L NaOH solution was dropped into the above solution under strong magnetic stirring until the PH of the solution was 9. The stirring of the solution was continued for 30 min. After the precipitate was washed three times by centrifugation, the dried product was calcined at 300°C for 2 h to give the hybrid Co_3O_4-NiO-seed.

Synthesis of NiO-Co_3O_4-P

Taking 0.5 mmol $NiCl_2$ and 1.5 mmol $CoCl_2$ and dissolved it together in 100 ml of deionized water, then, the 0.5 mol/L NaOH solution was dropped into the above solution under strong magnetic stirring until the PH of the solution was 9. The stirring of the solution was continued for 30 min. After the precipitate was washed three times by centrifugation, the dried product was calcined at 300°C for 2 h to give the sample of Co_3O_4-NiO-P.

Catalyst Characterization

The N_2 adsorption-desorption isotherms were measured by a TriStar 3000 volumetric adsorption analyzer at liquid nitrogen temperature. Each sample was sufficiently heated in a vacuum at 180°C for 6 h before measurement. The powder X-ray diffraction (XRD) was collected on a PANalytical Empyrean diffractometer instrument using Cu Kα radiation. The X-ray tube was operated at 45 kV and 40 mA. Raman spectroscopy was based on Renishaw Invia + Reflex Raman spectrometer, equipped with a CCD detector, and the excitation line wavelength was 514 nm. The H_2-Temperature programmed reduction (H_2-TPR) and $^{18}O_2$ isotopel labeling experiment were carried out in a BIAODE PCA-1200 adsorption

instrument equipped with online mass spectrometry (OmniStar/ThermoStar). In a typical H_2-TPR test, 50 mg catalyst was pretreated in Ar (50 mL/min) at 300°C for 1 h. After waiting for the oven to cool down to room temperature, the catalyst was re-heated in a flow of 5 vol.% H_2/Ar (20 mL/min) from 50°C to 600°C at a heating rate of 10°C /min. In the $^{18}O_2$ isotope labeling experiment, the NiO-Co_3O_4-seed was pretreated at 300°C under a flow of Ar for 60 min to remove the surface adsorbed oxygen. After the oven was cooled down to 280°C, the 5 vol.% CH_4/Ar was inducted into the reaction. After the reaction for 0.5 h, Ar gas was immediately switched to remove the residual CH_4 gas. Once the baseline in the mass spectrometer was steady, the reaction gas (5 vol.% CH_4/Ar with 1000 ppm $^{18}O_2$) was used. During the whole test, the outlet concentration of $C^{16}O_2$ (m/z = 44), $C^{16}O^{18}O$ (m/z= 46), and $C^{18}O^{18}O$ (m/z=48) were monitored by the on-line Mass Spectrometer. Transmission electron microscopy (TEM) images were recorded using a Talos L120C G2 electron microscope. Scanning Transmission Electron Microscopy (STEM) images and the Cs-corrected HRTEM images were analyzed on an FEI-Titan Cubed Themis G2 300 with double aberration correctors. X–ray photoelectron spectroscopy (XPS) was performed on a PHI 5000 Versa Probe X–ray photoelectron spectrometer equipped with Al Karadiation (1486.6 eV).

Activity evaluation

The low-temperature CH_4 catalytic combustion reaction was tested in a U-shaped quartz tube reactor with an inner diameter of 4 mm (D). Before the test, the 30 mg catalyst powder was diluted with 100 mg silica sand and then screened with 60-80 mesh size. Typically, the flow rate of reaction gas (1 vol.% CH_4 in dry air) was controlled at 20 mL/min by a mass flow controller. The outlet concentrations of CH_4/CO_2 were analyzed by an online gas chromatograph (GC2060, Shanghai Ruimin Instrument Co., Ltd).

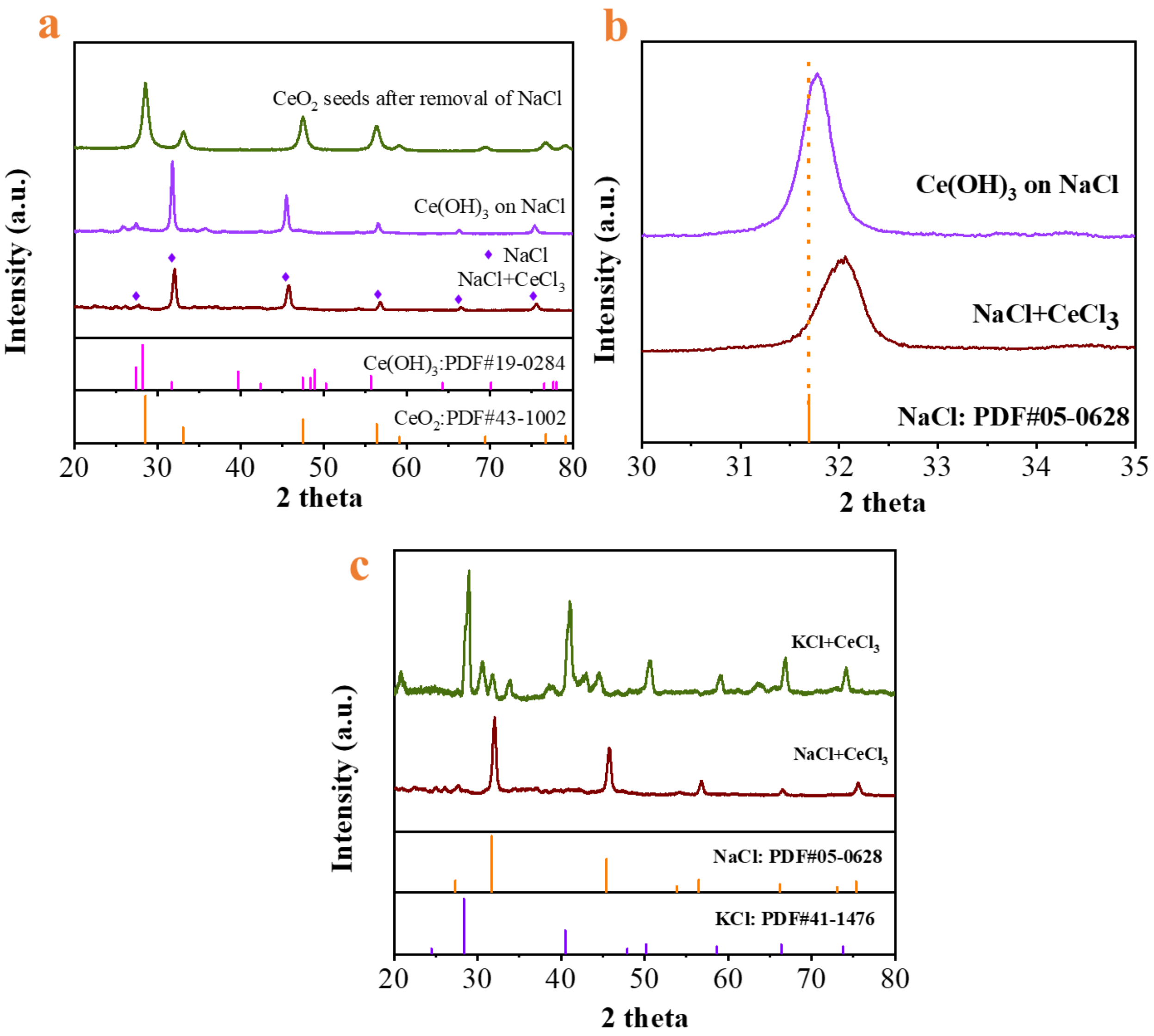

Figure S1. (a) XRD patterns of the intermediate products of ball-milling NaCl and $CeCl_3$. (b) the magnified XRD patterns of NaCl-$CeCl_3$ and $Ce(OH)_3/CeCl_3$/NaCl. (c) XRD patterns of the mixture by ball milling $CeCl_3$ and alkali metal salts (NaCl and KCl).

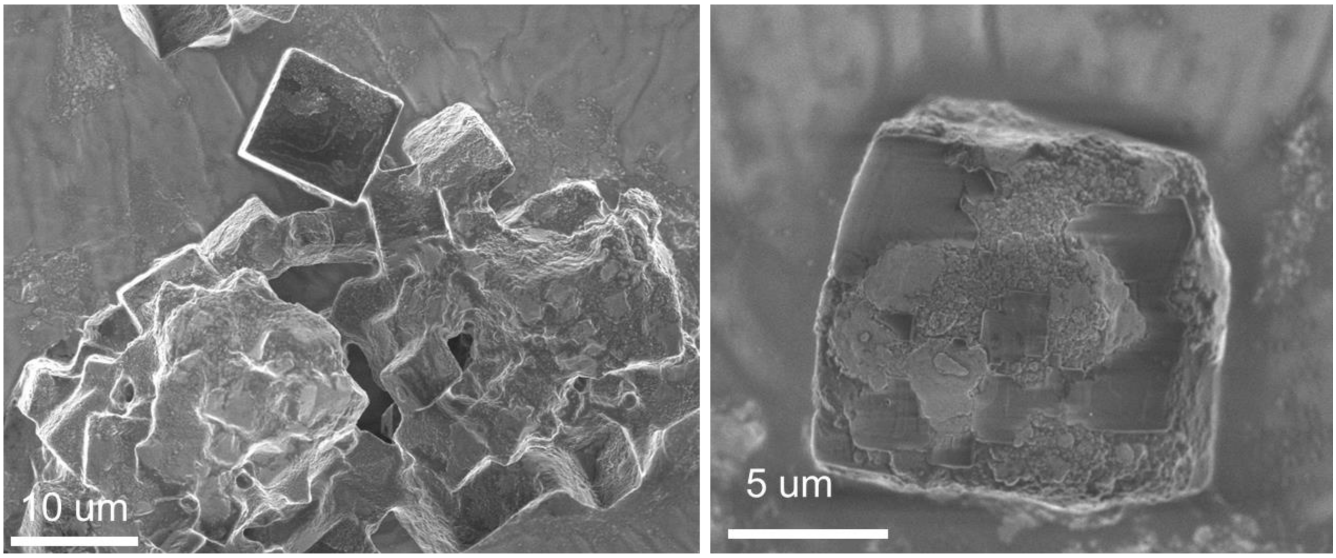

Figure S2. The SEM images of $CeCl_3$-NaCl after the first ball-milling.

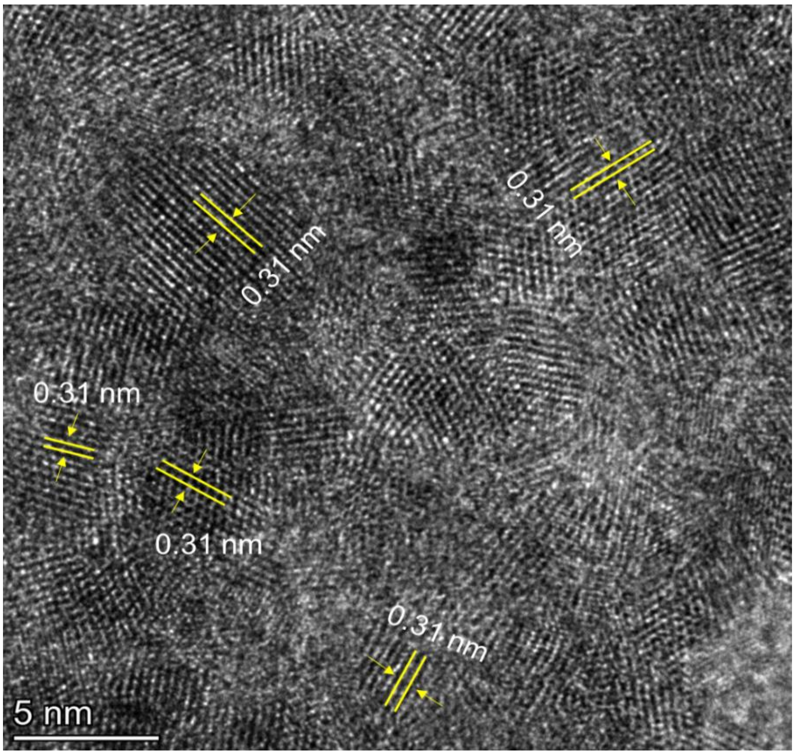

Figure S3. The HRTEM of CeO_2 seeds.

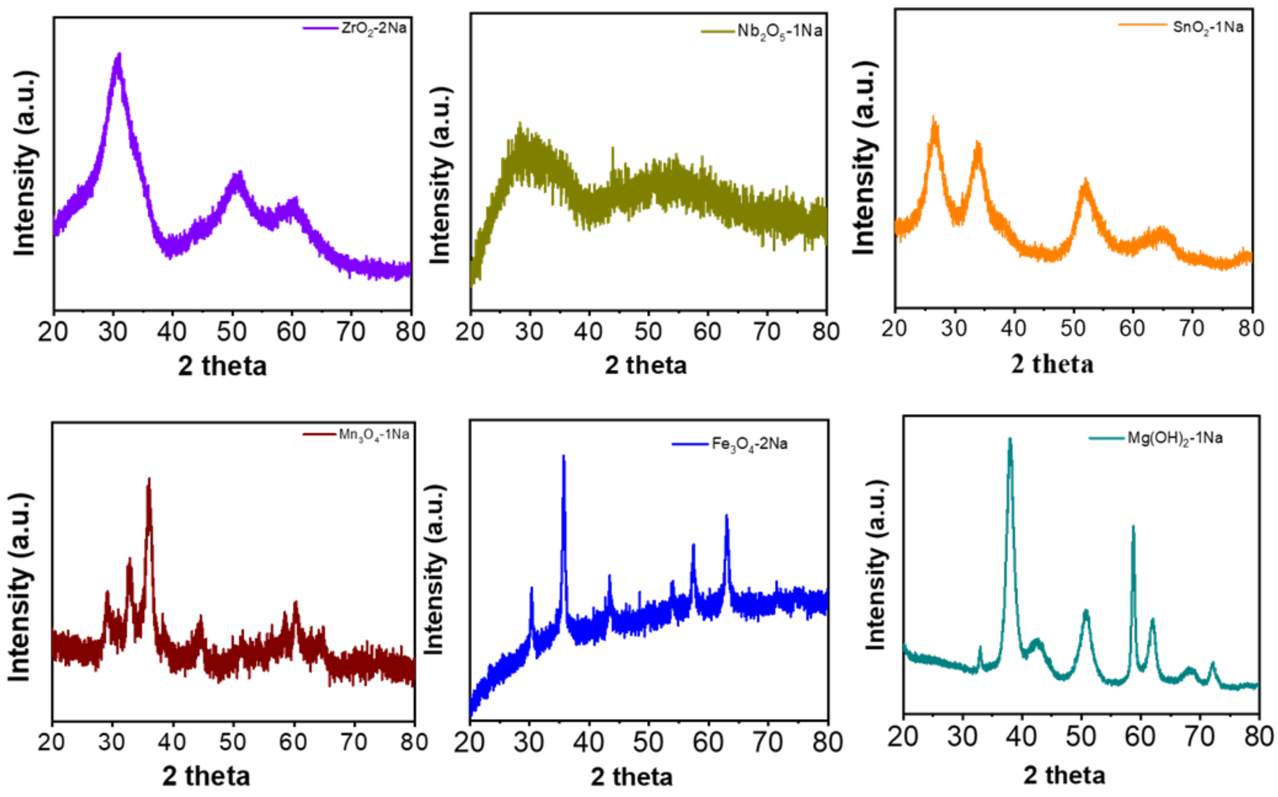

Figure S4. XRD patterns of the prepared TMOs.

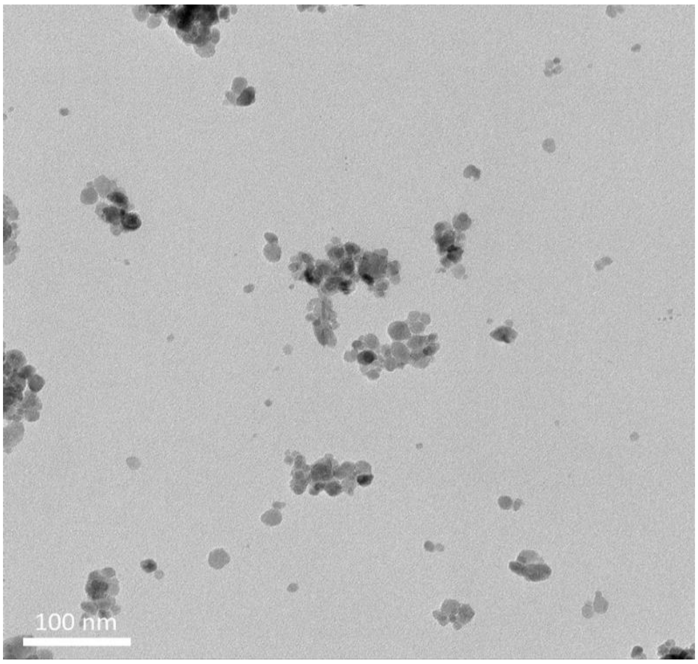

Figure S5. TEM image of Mn_3O_4 seeds.

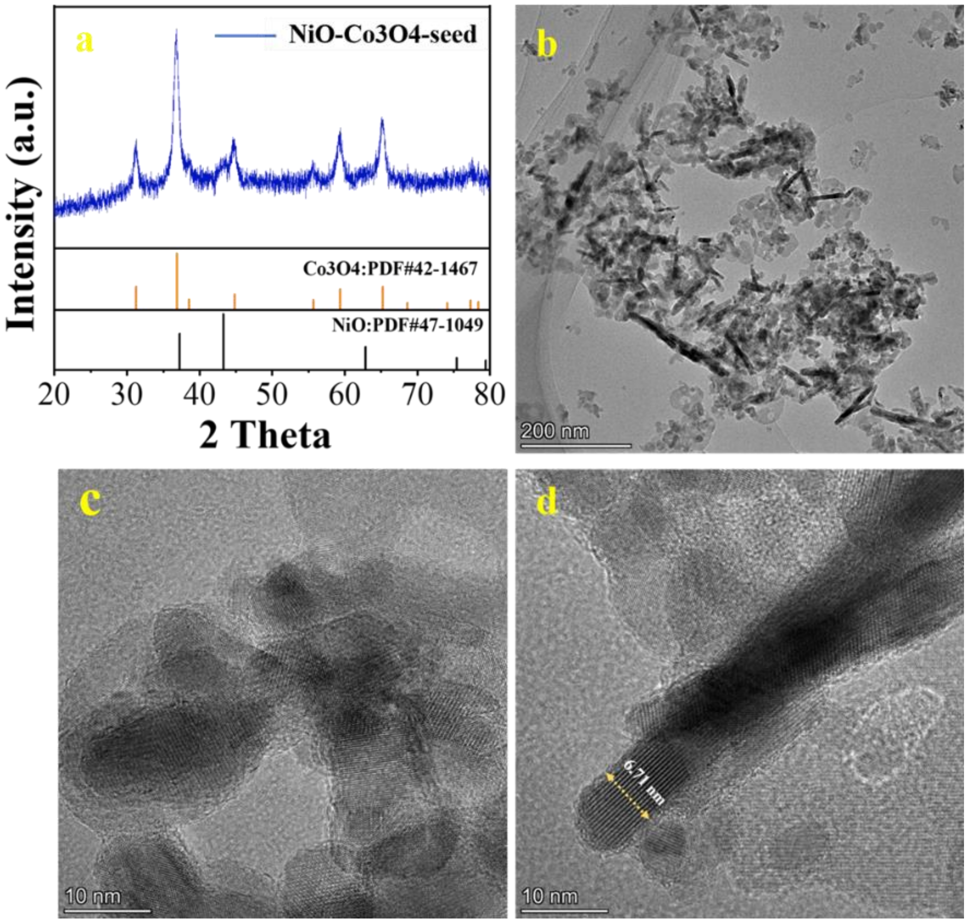

Figure S6. (a) XRD pattern of the spent NiO-Co_3O_4-seeds. (b-d) TEM and HRTEM image of the spent NiO-Co_3O_4-seeds.

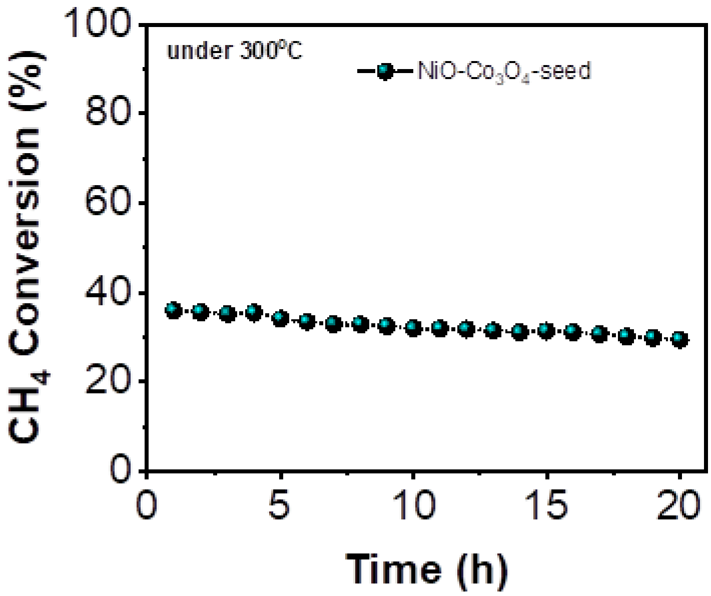

Figure S7. The stability testing of NiO-Co_3O_4-seed under 300℃.

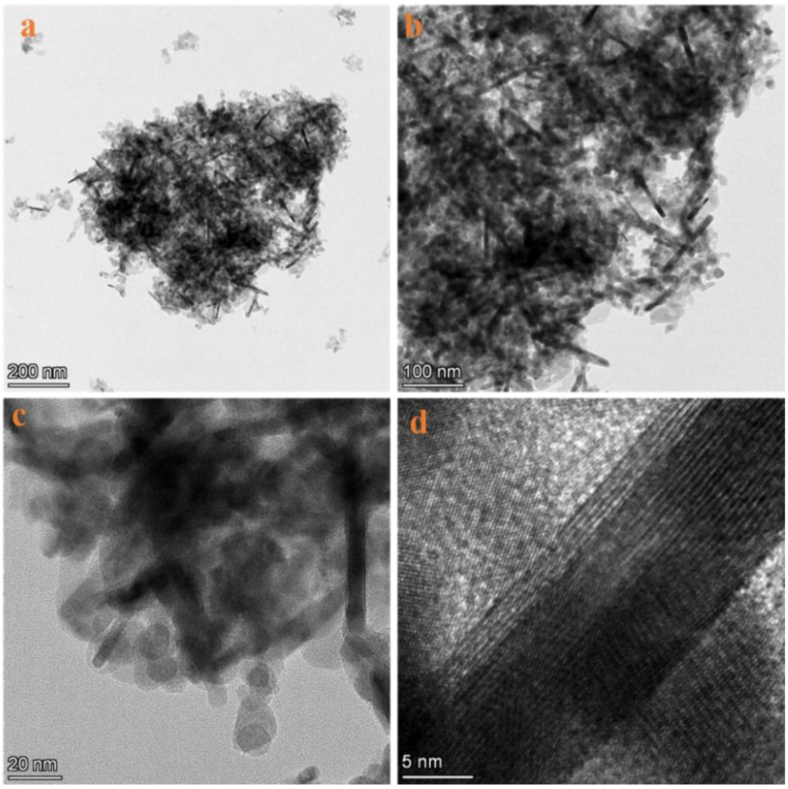

Figure S8. The TEM images of NiO-Co_3O_4-seed after 20 h stability testing.

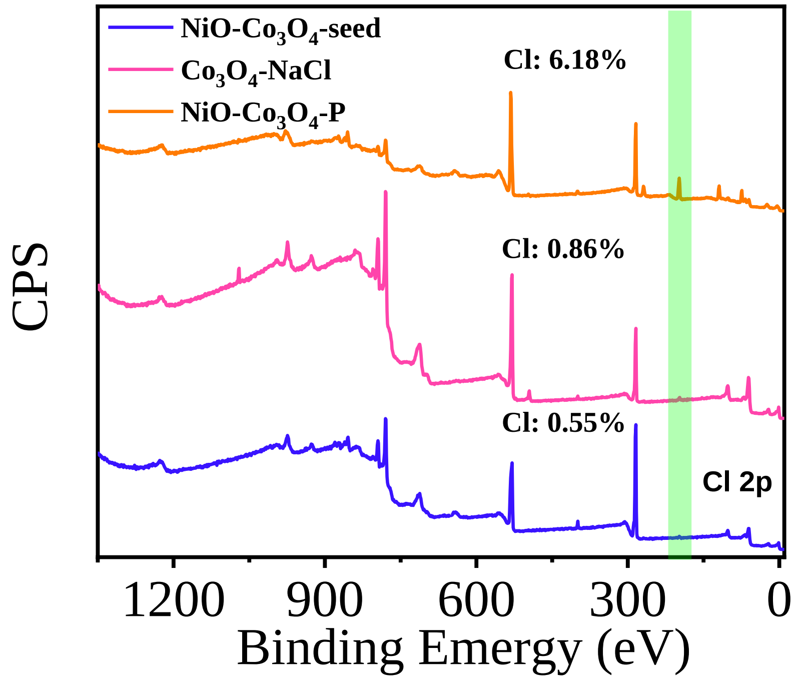

Figure S9. The XPS surveys of NiO-Co_3O_4-seed, NiO-Co_3O_4-P and Co_3O_4-NaCl.

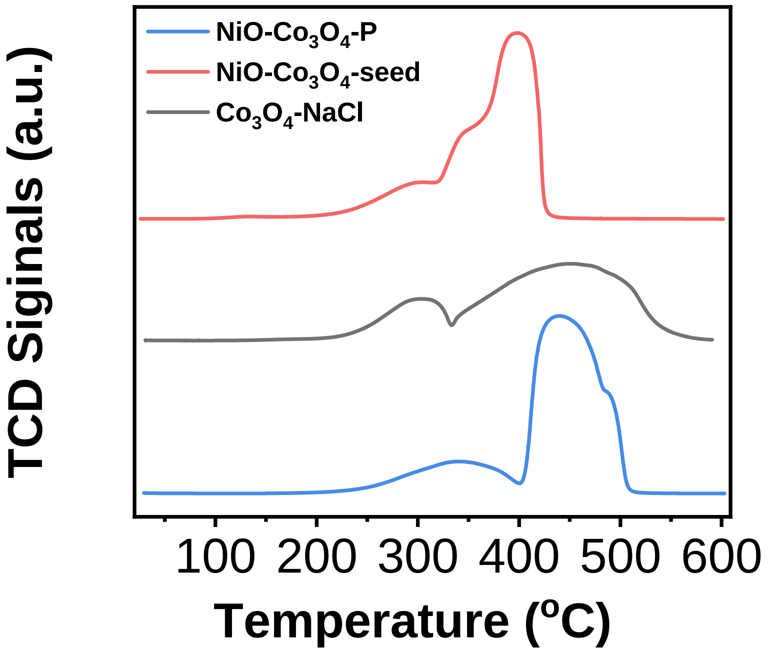

Figure S10. H_2-TPR curves of NiO-Co_3O_4-seed, NiO-Co_3O_4-P and Co_3O_4-NaCl.

Table S1. Comparison of the BET SSAs of TMOs with literature.

Sample	BET SSA	Crystalline (yes or no)	Using unrecoverable template (yes or no)	Raw material	source
CeO_2-2Na	189	yes	no	$CeCl_3$	This work
CeO_2	180	yes	Yes (Pluronic-123)	$Ce(NO_3)_3{\cdot}6H_2O$	[1]
CeO_2	126	yes	Yes (Pluronic-123)	$Ce(NO_3)_3{\cdot}6H_2O$	[2]
ZrO_2-2Na	332	yes	no	$ZrCl_3$	This work
ZrO_2	150	yes	Yes(P123)	$Zr(OBu)_4$	[1]
ZrO_2	422	-	Yes (polyethylenimine)	$Zr(OBu)_4$	[3]
SnO_2-1Na	276	yes	no	$SnCl_2$	This work
SnO_2	174	-	Yes (polyethylenimine)	Tin(II) ethoxide	[3]
SnO_2	204	yes	no	$Sn(OPr)_4$	[4]
Nb_2O_5-1Na	495	no	no	$NbCl_5$	This work
Nb_2O_5	279	yes	Yes (PEO-*b*-PS copolymer)	$NbCl_5$	[5]
Nb_2O_5	67	yes	Yes (polyethylenimine)	Nb(v) ethoxide	[6]
Fe_3O_4-1Na	212	yes	no	$FeCl_3$	This work
Fe_3O_4	127	-	Yes (Pluronic-123)	$Fe(NO_3)_3{\cdot}6H_2O$	[7]
Fe_3O_4	337	-	Yes (Pluronic-123)	$Fe(NO3)_3{\cdot}6H_2O$	[8]
Mn_3O_4-1Na	147	yes	no	$MnCl_2$	This work
Mn_3O_4	138	yes	Yes (CTAB)	$KMnO_4$	[9]
Mn_3O_4	55	yes	Yes(P123)	$Mn(ac)_2$	[10]
$Mg(OH)_2$-1Na	186	yes	no	$MgCl_2$	This work
$Mg(OH)_2$	92	yes	no	$Mg(NO_3)_2$	[11]

References

[1] G. Li, S. Dissanayake, S.-L. Suib, D. -E. Resasco, *Appl. Catal. B Environ.* **2020**, *26*, 118373.
[2] Y. Wang, X. Bai, F. Wang, S. Kang, C. Yin, X. Li, *J. Hazard. Mater.* **2019**,372,69-76,
[3] H. Xiong, T. Gao, K. Li, Y. Liu, Y. Ma, J. Liu, Z.-A. Qiao, S. Song, S. Dai, *Adv. Sci*. **2019**, 6, 1801543.
[4] V. Etacheri, G.-A. Seisenbaeva, J. Caruthers, G. Daniel, J.-M. Nedelec, V. G. Kessler, V. G. Pol. *Adv. Energy Mater.* **2015**. *5*,1401289.
[5] W. Luo, Y. Li, J. Dong, J. Wei, J. Xu, Y. Deng, and D. Zhao. *Angew. Chem. Int. Ed*. **2013**, *52*. 10505-10510.
[6] H. Xiong, H. Zhou, G. Sun, Z. Liu, L. Zhang, L. Zhang, F. Du, Z.-A. Qiao, S. Dai. *Angew. Chem. Int. Ed.* **2020**, *59*, 11053.
[7] D. Feng, T.-N. Gao, M.-H. Fan, A. Li., K.-Q. Li, T. Wang, Q.-S. Huo, Z.-A. Qiao. *NPG Asia Mater*. **2018**, *10*, 800–809.
[8] W.- R.K. Thalgaspitiya, T.-K. Kapuge, D. Rathnayake, J.-K. He, W.-S. Willis, S.-L. Suib, *Mater. Today*, **2020**, *35*, 50-68.
[9] S. Li, L.-L. Yu, R.-B. Li, J. Fan, J.-T. Zhao, *Energy Storage Mater.*, **2018**,*11*, 176-183.
[10] A.-Q. W, H. Wang, H. Deng, S. Wang, W. Shi, Z.-X. Yi, R.-L. Qiu, K. Yan, *Appl. Catal. B. Environ.* **2019**, *248*, 298-308,
[11] P.-S. Das, A. Dey, A.-K. Mandal, N. Dey, A.-K. Mukhopadhyay. *J. Adv. Ceram*. **2013**, *2*,173–179.

Author Contributions

The manuscript was written through the contributions of all authors. All authors have approved the final version of the manuscript.
